# Supplementary material for: The landscape of somatic mutation in sporadic Chinese colorectal cancer
Source: Oncotarget. 2018 Jun 8;9(44):27412–22. doi: 10.18632/oncotarget.25287 (PMC6007951; doi:10.18632/oncotarget.25287)
Supplement: Supplementary file 1 [file oncotarget-09-27412-s001.pdf]

The landscape of somatic mutation in sporadic Chinese colorectal cancer

SUPPLEMENTARY MATERIALS

Appendix A clustering of mutation spectrums

We performed log ratio transformation of the six mutation type data, and then used hierarchical clustering with Manhattan distance and centroid linkage for clustering. Silhouette plot is utilized to choose the best number of clusters on the basis of average silhouette width. The average silhouette plot of Chinese data is maximized when the number of clusters equals to 2 (Figure 1). The clustering results of TCGA data also indicate two clusters (Figure 2). The clustering results indicate that samples in Cluster 1 correspond perfectly to hypermutated samples. TCGA data also shows similar pattern and all of 33 the samples in Cluster 2 are hypermutated samples, only 4 hypermutated samples reside in Cluster 1. The results of Chinese and TCGA data are shown in Figures 3 and 4.

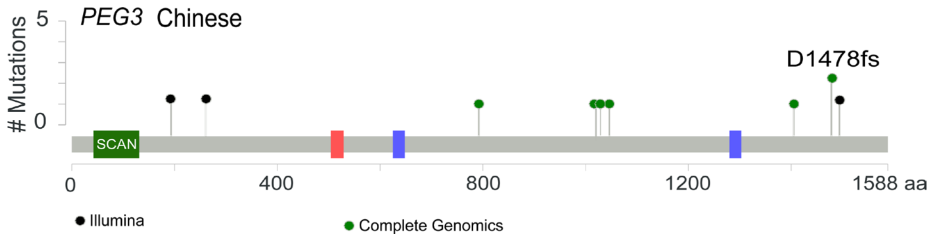

Supplementary Figure 1: Illustration of somatic mutations on PEG3.

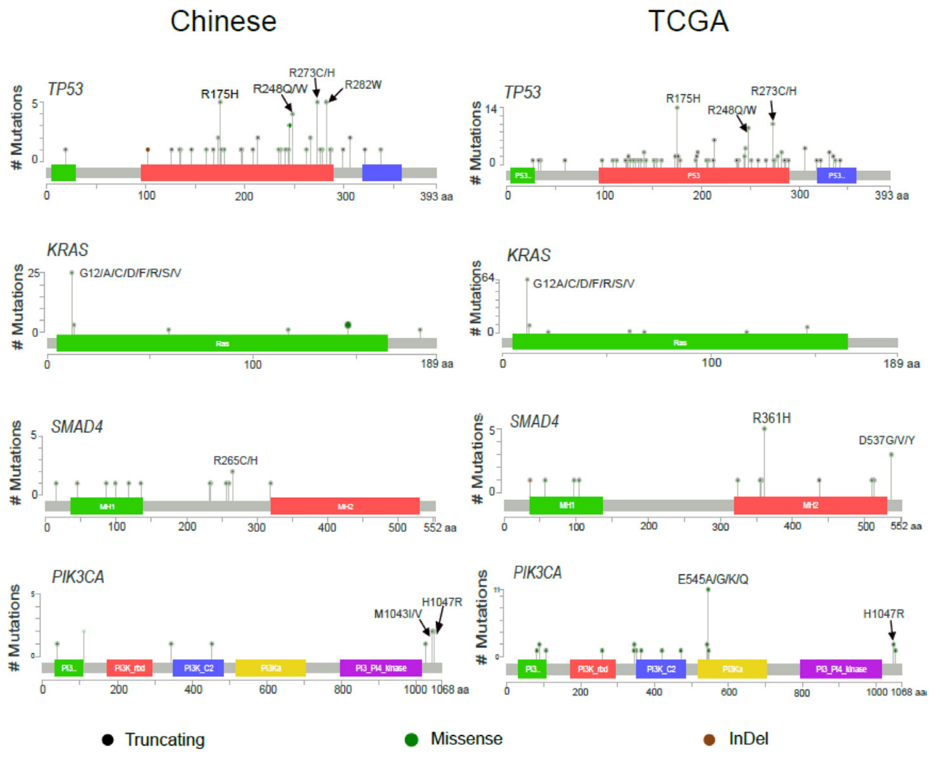

Supplementary Figure 2: Illustration of somatic mutations on canonical CRC genes.

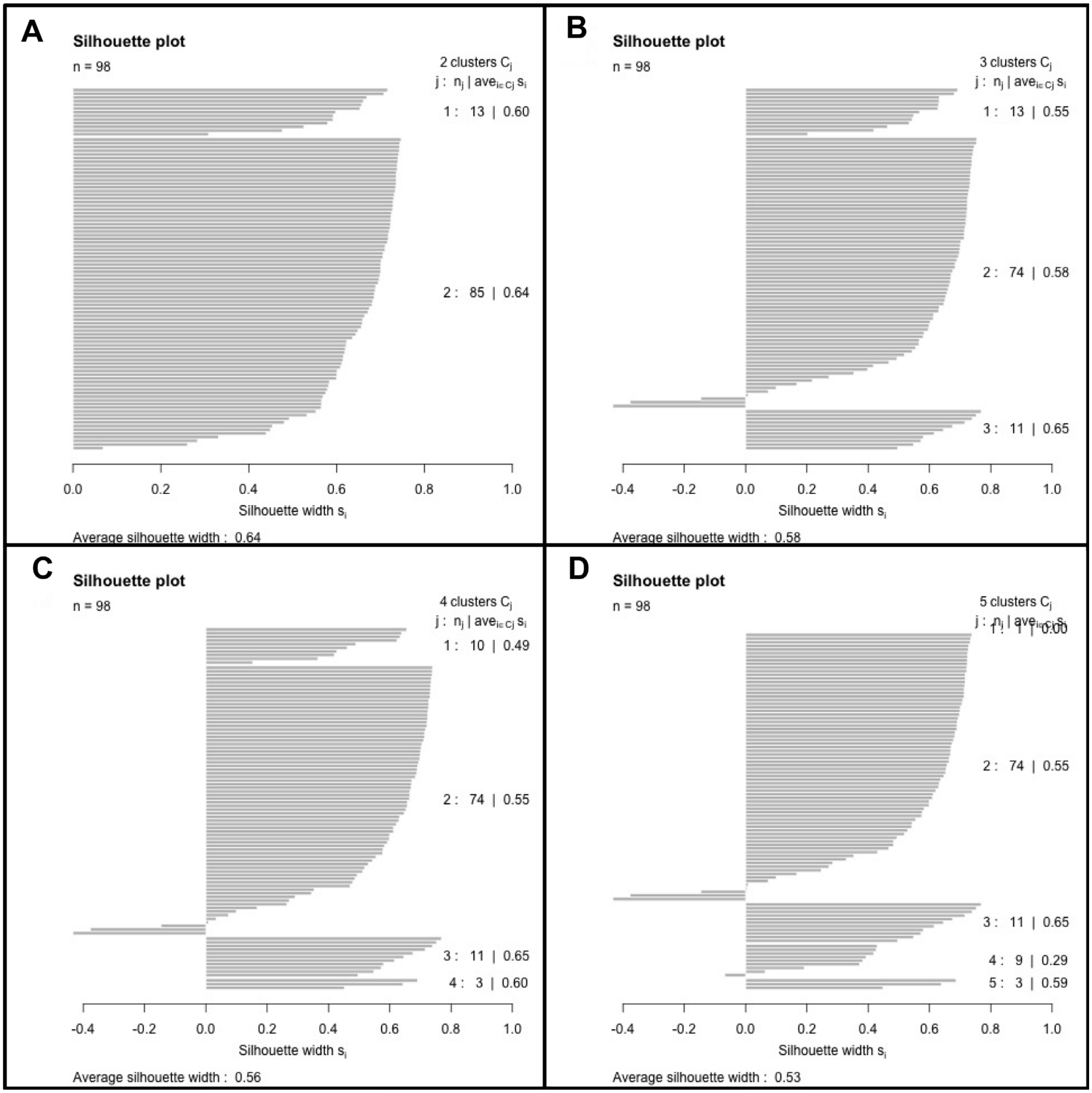

Supplementary Appendix 1: Silhouette plot of the Chinese data. (**A–D**) represent silhouette plot of 2, 3, 4 and 5 clusters of Chinese CRC data based on six mutation types.

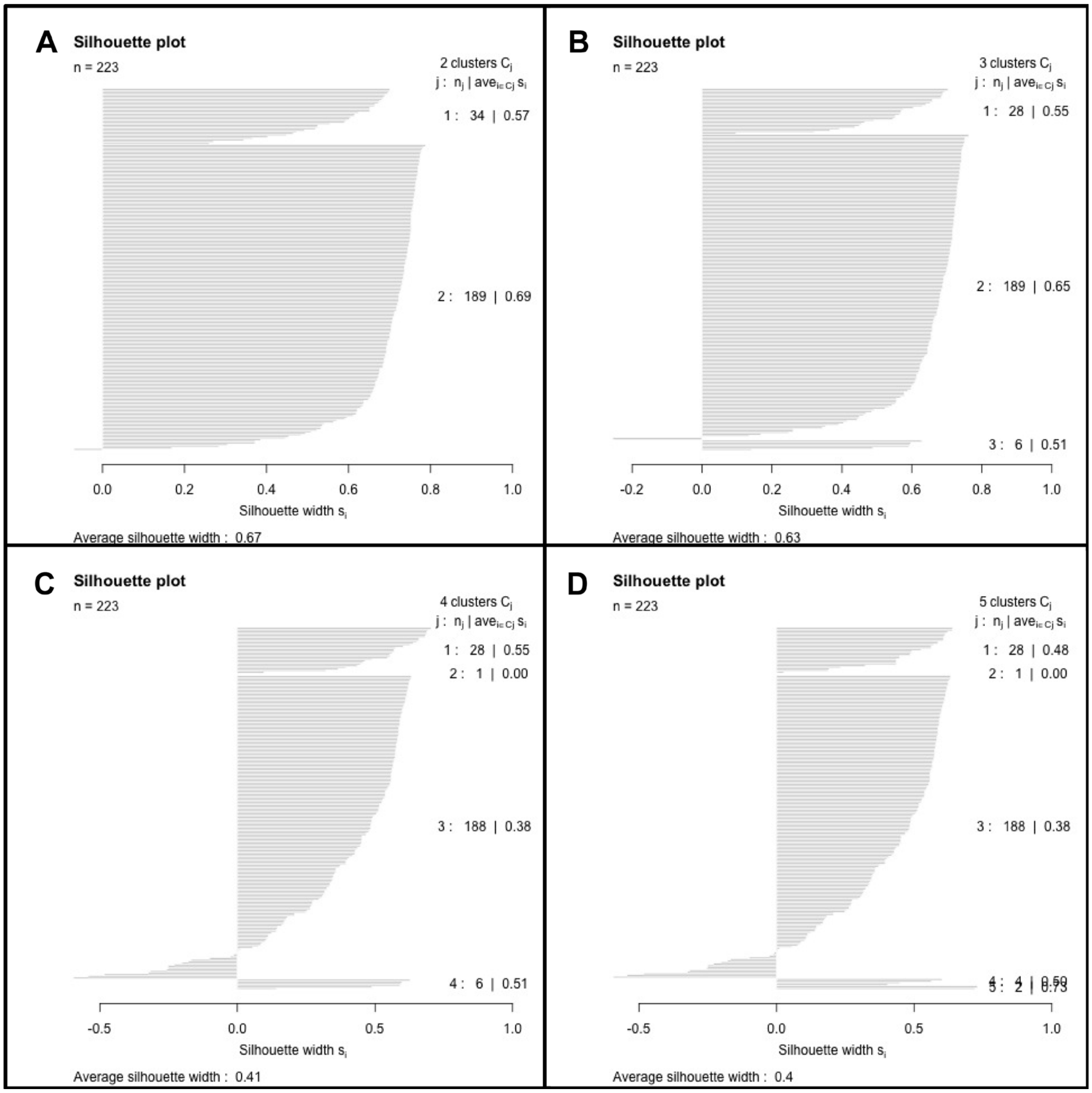

Supplementary Appendix 2: Silhouette plot of the TCGA data. (**A**–**D**) represent silhouette plot of 2, 3, 4 and 5 clusters of TCGA CRC data based on six mutation types.

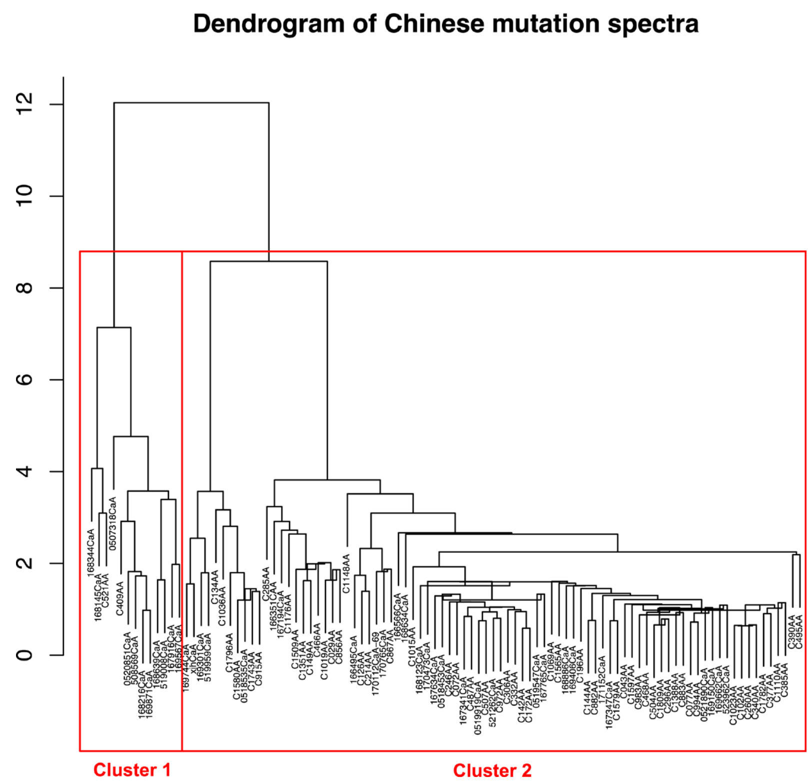

Supplementary Appendix 3: Clustering results of Chinese data.

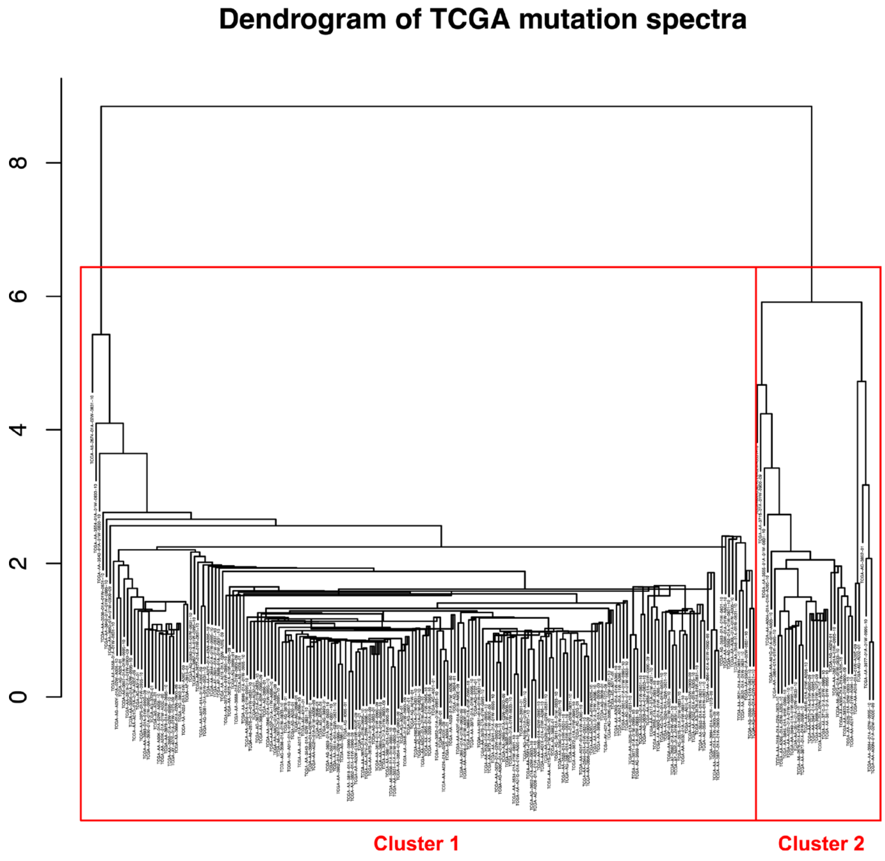

Supplementary Appendix 4: Silhouette plot of the TCGA data.

Supplementary Table 1: Clinicopathologic Features of the Patients and Tumors. See Supplementary_Table_1

Supplementary Table 2: Distribution of sequencing depth and coverages in 98 human colorectal cancer exomes. See Supplementary_Table_2

Supplementary Table 3: Somatic SNVs detected in tumors and matched controls. See Supplementary_Table_3

Supplementary Table 4: Somatic InDels detected in tumors and matched controls. See Supplementary_Table_4

Supplementary Table 5: Somatic mutated genes in hypermutated tumors. See Supplementary_Table_5

Supplementary Table 6 : Significantly mutated genes in regularly mutated tumors. See Supplementary_Table_6

Supplementary Table 7: Significantly mutated genes in regularly mutated tumors (ILMN). See Supplementary_Table_7

Supplementary Table 8: Significantly mutated genes in regularly mutated tumors (CG). See Supplementary_Table_8

Supplementary Table 9: Clinical characteristics association with five canonical CRC genes in TCGA data

		TP53		APC		KRAS		PIK3CA		PEG3	
		Mutation	*p*-value	Mutation	*p*-value	Mutation	*p*-value	Mutation	*p*-value	Mutation	*p*-value
**Age**	**<=45**	4 (66.7%)	1.000	2 (33.3%)	0.020	1 (16.7%)	0.223	0 (0%)	1.000	0 (0%)	1.000
	**>45**	107 (59.1%)		145 (80.1%)		84 (46.4%)		25 (13.8%)		5 (2.8%)	
**Sex**	**Male**	61 (59.2%)	1.000	84 (81.6%)	0.288	44 (42.7%)	0.461	13 (12.6%)	0.830	2 (1.9%)	0.658
	**Female**	50 (59.5%)		63 (75%)		41 (48.8%)		12 (14.3%)		3 (3.6%)	
**TNM stage**	**I+II**	58 (55.2%)	0.198	84 (80%)	0.831	53 (50.5%)	0.256	19 (18.1%)	0.066	4 (3.8%)	0.833
	**III**	35 (70%)		38 (76%)		21 (42%)		5 (10%)		1 (2%)	
	**IV**	18 (56.3%)		25 (78.1%)		11 (34.4%)		1 (3.1%)		0 (0%)	
**Lymph node**	**Positive**	60 (56.1%)	0.297	85 (79.4%)	0.857	54 (50.5%)	0.138	19 (17.8%)	0.051	4 (3.7%)	0.395
	**Negative**	51 (63.8%)		62 (77.5%)		31 (38.8%)		6 (7.5%)		1 (1.3%)	
**Tumor sites**	**Left colon**	50 (64.9%)	0.109	59 (76.6%)	0.316	24 (31.2%)	0.005	6 (7.8%)	0.086	0 (0%)	0.155
	**Right colon**	19 (46.3%)		30 (73.2%)		24 (58.5%)		9 (22%)		2 (4.9%)	
	**Rectum**	39 (65%)		51 (85%)		32 (53.3%)		6 (10%)		2 (3.3%)	
